# Effects of Danshen tablets on pharmacokinetics of amlodipine in rats

**DOI:** 10.1080/13880209.2019.1604768

**Published:** 2019-05-06

**Authors:** Haixia Zhang, Xiuyuan Han, Yiqing Li, Hangao Li, Xichun Guo

**Affiliations:** Department of Pharmacy, Affiliated Hospital of Weifang Medical University, Weifang, PR China

**Keywords:** Herb–drug interaction, CYP3A4, metabolism

## Abstract

**Context:** Danshen tablets (DST), an effective traditional Chinese multi-herbal formula, are often combined with amlodipine (ALDP) for treating coronary heart disease.

**Objective:** This study investigated the effects of DST on the pharmacokinetics of ALDP and the potential mechanism.

**Materials and methods:** The pharmacokinetics of ALDP (1 mg/kg) in male Sprague–Dawley rats (*n* = 6), with or without pretreatment of DST (100 mg/kg for 7 d), were investigated using LC-MS/MS. The effects of DST on the metabolic stability of ALDP were also investigated using rat liver microsomes (RLM).

**Results:** The results indicated that *C*_max_ (16.25 ± 2.65 *vs.* 22.79 ± 2.35 ng/ml), AUC_(0–_*_t_*_)_ (222.87 ± 59.95 *vs.* 468.32 ± 69.87 n gh/ml), and *t*_1/2_ (10.60 ± 1.05 *vs.* 14.15 ± 1.59 h) decreased significantly when DST and ALDP were co-administered, which suggested that DST might influence the pharmacokinetic behaviour of ALDP when they are co-administered. The metabolic stability of ALDP was also decreased (23.6 ± 4.7 *vs.* 38.9 ± 5.2) with the pretreatment of DST.

**Discussion and conclusions:** This study indicated that DST could accelerate the metabolism of ALDP in RLM and change the pharmacokinetic behaviours of ALDP. Accordingly, these results showed that the herb–drug interaction between DST and ALDP might occur when they were co-administered. Therefore, the clinical dose of ALDP should be increased when DST and ALDP are co-administered.

## Introduction

Amlodipine (ALDP) is a dihydropyridine calcium-channel blocker widely used for the treatment of hypertension and ischemic heart disease in the clinic (Adake et al. 2015; Agrawal et al. 2015; Mannemala and Nagarajan 2015; Naito et al. 2015; Feldman et al. 2016; Khodadoustan et al. 2017; Oh et al. 2017). ALDP is a substrate of CYP enzymes, and therefore, modulation of CYP activities may cause significant changes in the pharmacokinetic profiles of ALDP (Ryu et al. 2014; Zhu et al. 2014; Naito et al. 2015; Wang, Ouyang, et al. 2016). Lee et al. (2011) have reported that telaprevir, a potent inhibitor of both CYP3A4, increases the mean area under the curve (AUC) and the mean half-life of ALDP when the two drugs were co-administered. Glesby et al. (2005) also reported that indinavir and ritonavir could increase the median ALDP AUC_0–24_ by 90% when these drugs are co-administered. Therefore, drugs which inhibit the activity of CYP3A4 might affect the pharmacokinetic profiles of ALDP when they were co-administered (Hsiao et al. 2015; Zheng et al. 2016).

Danshen tablets (DST), an effective traditional Chinese multi-herbal formula, are widely used in treating cardiovascular diseases (Shi et al. 2016; Yin et al. 2016; Zhang et al. 2016; Yao et al. 2017). As we know, ALDP and DST are often simultaneously used for treating coronary heart disease in clinic in China. However, it is unknown whether there is an interaction between ALDP and DST. A better understanding of the pharmacokinetic interaction between DST and ALDP would help facilitate the design of rational dosage regimens and avoiding the occurrence of adverse reactions (Yang et al. 2011).

In this study, the potential herb–drug interactions of DST with ALDP were systematically investigated. The *in vivo* pharmacokinetics of ALDP in rats, with or without pretreatment with DST, were investigated using a sensitive and reliable LC-MS/MS method. The effects of DST on the metabolic stability of ALDP were also determined using rat liver microsomes (RLM).

## Materials and methods

### Materials and reagents

Standards of ALDP (purity >98%) and simvastatin (purity >98%) was purchased from the National Institute for the Control of Pharmaceutical and Biological Products (Beijing, China). Pooled RLM were purchased from BD Biosciences Discovery Labware (Woburn, MA). DST was purchased from Guangdong Baiyunshan Pharmaceutical Co., LTD (Guangzhou, China). Acetonitrile and methanol were purchased from Fisher Scientific (Fair Lawn, NJ). Ultrapure water was prepared with a Milli-Q water purification system (Millipore, Billerica, MA). All other chemicals were of analytical grade or better.

### Animals

This animal experimental protocol was approved by Experimental Animal Centre of the Weifang Medical University (Weifang, China). Male Sprague–Dawley rats weighing 220–250 g were supplied by Sino-British Sippr/BK Lab Animal Ltd (Shanghai, China). The rats were maintained in air-conditioned animal quarters at 22 ± 2 °C and 50 ± 10% relative humidity. Water and food were allowed *ad libitum*. The animals were acclimatized to the facilities for 5 d and then fasted with free access to water for 12 h prior to each experiment.

### Instrumentation and conditions

The analysis was performed on an Agilent 1290 series liquid chromatography system as previously reported (Liu et al. 2018) (Agilent Technologies, Palo Alto, CA) that included a binary pump, an on-line vacuum degasser, a surveyor autosampling system, a column temperature controller, and an Agilent 6460 triple-quadrupole mass spectrometer (Agilent Technologies, CA) with Turbo Ion spray, which is connected to the liquid chromatography system. The Agilent Mass Hunter version B 4.0 software (Agilent Technologies, CA) was used to control the equipment and for data acquisition. The chromatographic analysis of ALDP was performed on a Waters X-Bridge C18 column (3.0 × 100 mm, i.d.; 3.5 μm, USA) at room temperature. The mobile phase was water (containing 0.1% formic acid) and acetonitrile (30:70, v:v) at a flow rate of 0.4 ml/min.

The mass scan mode was a positive MRM mode. The precursor ion and product ion were *m/z* 559.2 → 440.2 for ALDP and *m/z* 419.9 → 199.4 for the simvastatin, respectively. The collision energy for ALDP and simvastatin were 30 and 25 eV, respectively. The MS/MS conditions were optimized as follows: fragmentor, 140 V; capillary voltage, 4 kV; nozzle voltage, 500 V; nebulizer gas pressure (N_2_), 40 psig; drying gas flow (N_2_), 10 l/min; gas temperature, 350 °C; sheath gas temperature, 400 °C; and sheath gas flow, 11 l/min.

### Pharmacokinetic study

For pharmacokinetic study *in vivo*, 12 rats were equally randomized to two groups, six rats in each group. The test group was pretreated with DST at a dose of 100 mg/kg/d for 7 d before the administration of ALDP. Next, ALDP were orally administered to rats by gavage at a dose of 1 mg/kg (Zhang et al. 2018). Blood samples (0.2 ml) were collected into a heparinized tube *via* the *oculi chorioideae* vein before drug administration and at 0.083, 0.25, 0.5, 1, 2, 3, 4, 6, 8, 12, 24, 36 and 48 h after drug administration. After centrifuging at 3500 rpm for 10 min, the supernatant was obtained and frozen at −40 °C until analysis.

### Pharmacokinetic analysis

The pharmacokinetic parameters were calculated using DAS version 3.0 pharmacokinetic software (Chinese Pharmacological Association, Anhui, China). Experimental values are expressed as mean ± SD. Statistical analysis of results obtained from clinical study was performed using Student’s paired *t-*test. Differences were considered statistically significant when *p* < 0.05.

### Inhibitory effects of DST on the metabolic stability of ALDP in rat liver microsomes

RLM were used to determine the metabolic rate of ALDP. The assay conditions and reaction mixtures were similar as reported previously (Qi et al. 2013; Qin et al. 2014). The reaction mixture was incubated at 37 °C for 5 min and then ALDP (100 μM) was added. The effects of DST on the metabolic rate of ALDP was investigated by adding 50 μg/ml of DST to RLM and preincubating for 30 min at 37 °C, and then ALDP (100 μM) was added. Additionally, the specific CYP3A4 inhibitor (verapmil, 50 μM) or inducer (rifampicin, 50 μM) were also used to investigate its effects on the metabolic rate of ALDP. Aliquots of 30 μl were collected from reaction volumes at 0, 1, 3, 5, 15, 30 and 60 min and 60 μl ice-cold acetonitrile was added to terminate the reaction, and then the sample preparation method was the same as the plasma sample preparation method and determined by LC-MS/MS.

The half-life (*t*_1/2_) *in vitro* was obtained using the equation: *t*_1/2_ *=* 0.693/*k*.

### Statistical analysis

Experimental values are expressed as mean ± SD. Statistical analysis of results obtained from clinical study was performed using Student’s paired *t-*test. Differences were considered statistically significant when *p* < 0.05. Statistical analysis was conducted using GraphPad Prism version 3.0 for Windows (GraphPad Software Inc., San Diego, CA).

## Results

### Pharmacokinetic study *in vivo*

**Figure 1.**
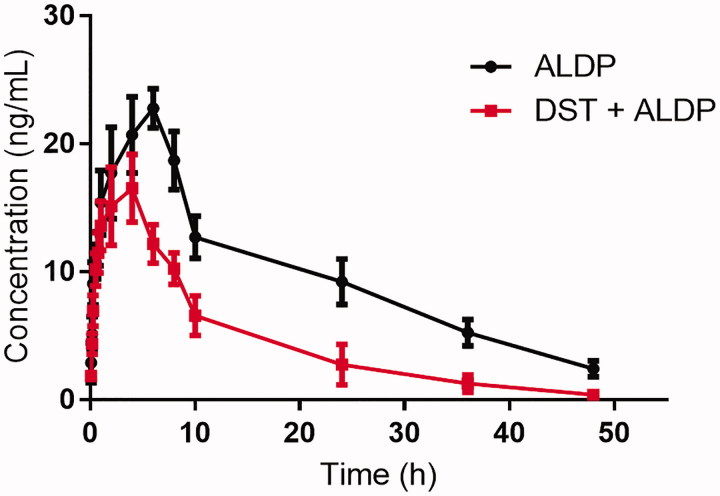
The mean concentration-time curves in male Sprague–Dawley rats (*n* = 6, mean ± SD) of ALDP (1 mg/kg) with or without pretreatment of DST (100 mg/kg for 7 d).

The mean plasma concentration-time curves of ALDP with or without pretreatment of DST are presented in Figure 1 and the pharmacokinetic parameters are shown in Table 1.

**Table 1.** Pharmacokinetic parameters of ALDP in male Sprague–Dawley rats (*n* = 6, Mean ± SD) following oral administration of ALDP alone (group A) and both ALDP and DST (group B).

Parameter	ALDP	
	Group A	Group B
*T*_max_ (h)	5.89 ± 0.56	4.02 ± 0.38*
*C*_max_ (μgl^−1^)	22.79 ± 2.35	16.25 ± 2.65*
*t*_1/2_ (h)	14.15 ± 1.59	10.60 ± 1.05*
AUC_(0–_*_t_*_)_ (μghl^−1^)	468.32 ± 69.87	222.87 ± 59.95*
CL (lh^−1^kg^−1^)	1.93 ± 0.32	4.55 ± 2.65*

*p < 0.05 indicate significant differences from the control.

The results indicated that the *C*_max_ (16.25 ± 2.65 *vs.* 22.79 ± 2.35 ng/ml), AUC_(0_*_–t_*_)_ (222.87 ± 59.95 *vs.* 468.32 ± 69.87 n gh/ml), and *t*_1/2_ (10.60 ± 1.05 *vs.* 14.15 ± 1.59 h) decreased significantly when DST and ALDP were co-administered, which suggested that DST might influence the pharmacokinetic behaviour when they are co-administered. These results suggested that the herb–drug interaction between ALDP and DST might occur when they are co-administered. As the plasma concentration of ALDP decreased when co-administered with DST, which suggested that the pharmacological activities of ALDP might be weakened, and therefore, the clinical dose of ALDP should be adjusted when they are used simultaneously. Salvianolic acid B is a major component isolated from Danshen and previous studies (Wang, Zhang, et al. 2016) have reported that salvianolic acid B could induce the activity of CYP3A4 in a concentration-dependent manner. As ALDP is a substrate of CYP3A4 enzymes, which is predominantly metabolized by CYP3A4 (Lee et al. 2015), and therefore, we infer that the herb–drug interaction between ALDP and DST may occur due to the effects of salvianolic acid B on the activity of CYP3A4.

### Inhibitory effects of DST on the metabolic stability of ALDP in rat liver microsomes

As we know, the metabolism of ALDP was mainly modulated by CYP3A4 enzymes and, therefore, in this research, the effects of DST on the metabolic stability of ALDP were further investigated in RLM *in vitro*. The metabolic stability of ALDP was 38.9 ± 5.2 min, while the metabolic stability was decreased in the presence of DST (23.6 ± 4.7 min) or rifampicin (15.2 ± 3.1 min). However, metabolic stability was increased in the presence of verapamil (57.6 ± 7.0 min). The results indicated that DST could accelerate the metabolism of ALDP in RLM and change the pharmacokinetic behaviours of ALDP.

## Discussion

The pharmacokinetic experiments showed that DST could decrease the system exposure of ALDP in rats. To investigate its potential mechanism, the metabolism clearance was also investigated using RLM, and the results revealed that DST could increase its metabolism clearance in rat liver through inducing the activity of CYP3A4. ALDP is metabolized predominantly *via* CYP3A4 enzyme, and therefore, co-administration of foods or drugs with influence on CYP3A4 enzymes may affect the pharmacokinetics of ALDP. Previous studies (Jia et al. 2018; Zhang et al. 2018) have also reported that drugs or herbs could affect the pharmacokinetics of ALDP through accepting the activity of CYP3A4 enzyme.

Therefore, this study’s results indicate that when the rats were pretreated with DST, the system exposure of ALDP would be decreased significantly. The results indicate that the herb–drug interaction between DST and ALDP might occur when they were co-administered. These changes could decrease ALDP efficacy, so it was suggested that the dosage should be adjusted if DST and ALDP are co-administered in the clinics.

In conclusion, the results indicated that DST could influence the pharmacokinetic behaviour ALDP when they are co-administered. DST could accelerate the metabolism of ALDP in RLM, and the metabolic stability of ALDP was decreased, which may be one of the reasons resulting in pharmacokinetic interactions when they were co-administered. Therefore, the clinical dose of ALDP should be increased when DST and ALDP are co-administered.
